# Normal and Pathological Placental Angiogenesis

**DOI:** 10.1155/2015/354359

**Published:** 2015-01-31

**Authors:** Nathalie Bardin, Padma Murthi, Nadia Alfaidy

**Affiliations:** ^1^Inserm UMR-S 1076, Faculté de Pharmacie, Aix-Marseille University, 27 Boulevard Jean Moulin, 13385 Marseille Cedex 05, France; ^2^Department of Obstetrics and Gynaecology (RWH), University of Melbourne, Parkville, VIC 3052, Australia; ^3^Inserm U1036, University Grenoble Alpes, CEA, 38054 Grenoble, France

Placental angiogenesis is a pivotal process that establishes fetomaternal circulation, ensures efficient maternofetal exchanges, plays a key mechanistic role in the elaboration of the placental villous tree, and contributes to the overall development of the placenta throughout pregnancy. Failure in these processes is tightly linked to the development of placental pathologies such as preeclampsia (PE), early pregnancy loss, and intrauterine growth restriction (IUGR). It is now well established that a close relationship exists between embryonic development and the degree of placental angiogenesis. A key discovery in the investigation of placental pathologies was the understanding that major phenotypes of PE are associated with dysregulation in angiogenic factors. During the last decade multiple new key angiogenic proteins have been reported to play crucial roles in placental angiogenesis; these include netrins, EG-VEGF (endocrine gland derived endothelial growth factor), and angiogenin. Furthermore, placental angiogenesis appears to also be regulated by specific microRNAs (miRNAs), deregulations of which have also been shown to be associated with pregnancy pathologies. By affecting placental angiogenesis, numerous insults have been shown to influence pregnancy outcomes. These include (i) oxidative stress, (ii) hyperglycemia, and (iii) failure in immune system adaptations to pregnancy, such as the presence of antiphospholipid antibodies (aPL) and/or soluble Mic (sMIC).

In the present scope authors reported new findings in the field of placental angiogenesis, discussed the advancements made in the diagnosis of pathologies reported to be associated with placental angiogenesis, and brought new insights into the processes of vasculogenesis and angiogenesis occurring throughout pregnancy in the placenta. More importantly, regulators of the key protagonists of vascular and angiogenic processes have been reported and their roles discussed.

The reviews by M. Dakouane-Giudicelli et al., N. Pavlov et al., and N. Alfaidy et al. reported the role of three newly discovered angiogenic placental factors, netrin-1 and netrin-4, angiogenin, and EG-VEGF, respectively. M. Dakouane-Giudicelli et al. highlighted the opposite role that netrin-1 and netrin-4 might play in normal and pathological pregnancies. Netrin-1 and netrin-4 have been found to be either proangiogenic or antiangiogenic factors in the human placenta. These opposite effects appear to be related to the endothelial cell phenotype studied and seem also to depend on the type of receptor to which each netrin binds.

N. Pavlov et al. demonstrated that the proangiogenic protein, angiogenin, might play a key role in placental blood vessel formation as well as in the cross talk between trophoblasts and endothelial cells. The review by N. Alfaidy et al. recapitulates EG-VEGF mediated-angiogenesis within the placenta and at the fetomaternal interface and proposes that its deregulation might contribute to the pathogenesis of several placental pathologies including IUGR and PE. More importantly, this article argues for EG-VEGF clinical relevance as a potential biomarker of the onset of pregnancy pathologies and discusses its potential usefulness for future therapeutic directions.

In a second set of reviews of the scope Dr. R. D. Pereira et al. reported the effect of oxidative stress on the expression of a number of transcription factors that are important in mediating angiogenesis and stressed that the understanding of how oxidative stress affects redox-sensitive transcription factors within the placenta may elucidate potential therapeutic targets to correct abnormal placental angiogenesis and function. In the same context, S. Cvitic et al. described the major regulators of placental angiogenesis and demonstrated how the diabetic environment* in utero* alters their expression.

In relation to immune placental pathologies that affect placental vascularization and angiogenesis, J. B. Haumonte et al. work suggests that detection of sMIC molecules in maternal plasma may constitute a hallmark of altered maternal immune functions associated with vascular disorders that contributes to poor pregnancy outcomes, notably by impairing NK-cell mediated production of IFN gamma, an essential cytokine favoring vascular modeling. In the same perspective, O. Paulmyer-Lacroix et al. highlighted the menace of antiphospholipid antibodies (aPL) positive patients on placental vascular development during implantation. These findings propose aPL assessment, in particular a*β*2GPI IgA antibodies, as a novel therapeutic strategy to improve pregnancy outcome.

Finally, M. Tsochandaridis et al. highlighted a major topic of interest for clinicians and biologists that concern maternal and fetal monitoring and their importance in predicting pregnancy complications. Her work has addressed the potential role of circulating as well as placental miRNAs that are associated with abnormal angiogenesis.

Overall the present scope on this special issue summarizes key aspects of placental angiogenesis during normal and pathological pregnancies. The scope will expand our knowledge in this field and allow better understanding of the associated mechanisms to placental angiogenesis.

*Nathalie Bardin* *Padma Murthi* *Nadia Alfaidy*

